# Too soon to worry? Longitudinal examination of financial planning for retirement among Spanish aged workers

**DOI:** 10.1371/journal.pone.0209434

**Published:** 2018-12-14

**Authors:** Francisco Palací, Irene Jiménez, Gabriela Topa

**Affiliations:** National Distance Education University (UNED), Department of Social and Organizational Psychology, Madrid, Spain; University of Nottingham School of MedicineD, UNITED KINGDOM

## Abstract

The present study analyzes the relationship between three distal antecedents—financial literacy, confidence in retirement, and economic well-being—and financial planning for retirement evaluated at two different times. We used longitudinal data with repeated measures of financial planning for retirement obtained from a sample (N = 269) of active Spanish workers aged 45–62 years. The results confirm that self-perceived financial knowledge, confidence in retirement, and economic well-being are associated with financial planning for retirement at three and six months. The stability of financial planning for retirement over time was a relevant finding in the present research, even though different measures have been employed in the two waves and financial planning decreases slightly at three months. While the first step of planning, at three months, has predictive power over the second, at six months, there are possible moderators in the relationship between financial planning for retirement at time 1 and time 2, which were not explored. The implications of the results both for financial education and Policy-makers are discussed. Future lines of research can explore these relationships including objective measures of income, as wealth accumulation.

## Introduction

Financial planning for retirement (hereinafter, FPR) is a complicated task for most people. Among other reasons, the difficulty lies in the fact that FPR requires thinking about the future and saving based on the anticipation of the needs one will have during retirement [1]. And at the same time, planning is a pressing task. In the past decade, the governments and policy makers insist on threats posed to public pension systems as the only guarantors of the well-being of retired people [2]. Despite this, many people do not save for retirement, even when the reforms of the Public Pensions System during 2011 and 2013 led to a progressive reduction of the average pension value, which has been estimated as 30% less between 2010 and 2050, at least in Spain. Finally, given that saving has no immediate effect, the persistence of FPR is another aspect that deserves special attention. Thus, proper financial planning requires individuals to maintain a sustained effort over time to meet their future personal needs [3]. It seems clear, therefore, that FPR is subject to the influence of a wide range of antecedents and, moreover, its temporary stability is questionable [4].

Therefore, this study intends to analyze the influence of distal antecedents on FPR, evaluated at two consecutive moments according to the model of Hershey, Jacobs-Lawson, and Austin [5]. The model proposes that FPR is determined by individuals' capacity, disposition, and opportunities to plan and save for retirement, as well as the interaction of these dimensions. Hence, we intend to examine the influence of self-perceived financial knowledge, confidence in retirement, and economic well-being in FPR, considered as distal antecedents. In addition, we will explore how a first measure of FPR (hereinafter, FPR1) mediates the relationship between these distal antecedents and a subsequent measure of FPR (hereinafter, FPR2).

In order to be able to provide informed recommendations to individuals, organizations, and governments, we need to deepen our understanding of the factors influencing FPR. This will allow us to offer suggestions that promote greater involvement of good planners or the development of compensatory strategies to correct negative situations among those who still do not plan.

### Financial planning for retirement (FPR)

For a clear majority of people, savings are the best guarantee of economic well-being for retirement. On another hand, those who do not save for this vital stage usually intend to rely on the pensions that social security will grant those [6]. However, some empirical studies indicate that confidence in the economic pensions that will be available upon retirement is becoming increasingly weaker [7], even in those countries that have built a solid welfare state in the last decades. But, despite this lack of confidence, many people do not save, or do not do so continuously over time, to provide sufficient financial support in old age [8].

For some time, the literature has been pointing out that people who plan their retirement have more positive attitudes towards this stage [9, 10]. In addition, people who have positive attitudes toward retirement are seeking more advice and guidance from professionals in the financial field [11]. Quantitative studies supported that the future time perspective is related to greater involvement in specific behaviors to achieve the goals that have been set [12, 13]. Thus, it can be said that those who have more positive attitudes towards their future as retirees will engage in stable FPR.

Previous research showed that the contributions of savings differ according to people's employment status and current professional stage [14]. In addition, predictions made by individuals about their future expenditures have a strong influence on savings and financial planning, as these predictions are a component of any planning [15]. Moreover, the spending predictions may act as an incentive or standard that promotes subsequent performance. However, it has been observed that predictions about future spending are often quite biased [16], and usually exaggeratedly optimistic. The findings of these studies, even though based on experiments, supported that positive or negative predictions can influence later behavior, despite that multiple cognitive variables could influence biased optimistic beliefs [17].

In relation to the stability of FPR, it has been found that parental financial socialization [18], as well as saving behavior in childhood, increase the likelihood of saving during adulthood [19]. In this sense, the *consistency paradox* can be applied to FPR, which establishes that past behavior is a good predictor of future behavior, although at any given time, behavior can change [20]. This would allow expecting the behavior of saving, once initiated, would show temporal consistency [21]. Based on this principle, we foresee that FPR, once initiated, will be sustained over time.

### Self-perceived financial knowledge

The literature emphasizes the relationship between cognitive abilities and financial behavior [22]. Survey studies support that people with greater numerical ability show more cautious financial behaviors [23]. Financial knowledge is the ability of individuals to understand and integrate financial information, influencing their ability to make decisions about financial aspects [24, 25]. In this line, it has been found that the ability of household members to make financial decisions will impact on long term economic well-being of households [26]. Several studies in recent years are pointing in the same direction, finding that people who have greater financial literacy plan the economic aspect of their retirement more and have greater economic well-being [3, 27].

The broader field of literature on financial literacy and retirement [5, 21], and more specifically the findings of Lusardi and Mitchell [28] have shown that financial literacy influences retirement planning in general and financial well-being during retirement [29]. In this sense, two kinds of financial knowledge measures can be distinguished. On the one hand, a more objective financial knowledge measure using elemental calculations has been recommended [29]. On the other hand, a subjective assessment of financial knowledge has also been used, as self-perceived financial knowledge. It consists on the confidence of the respondents on his/her knowledge on the topic. Following the later approach, we focused on self-perception of financial knowledge.

Precedent literature supported that perceptions of financial knowledge also have been positively associated with the level of confidence in retirement [30] and with saving behavior [31, 32, 33]. Moreover, recent works in different countries are still pointing to the positive relationship between financial knowledge and FPR [34, 35]. Thus, direct relations between financial knowledge and FPR have already been explored cross-sectionally [36], but there are few works showing how a first measure of FPR may mediate the relationship between financial knowledge and a second measure of FPR. Thus, we propose the following working hypothesis:

Hypothesis 1: Self -perceived Financial knowledge will predict FPR2, and this relationship will be mediated by FPR1.

### Confidence in retirement

In addition to cognitive antecedents, capabilities to manage finances seem to be influenced by motivational and emotional variables, as retirement confidence [37]. Greater perceived control of one's personal economic future and with greater confidence in the concrete possibility of achieving desired goals promotes more concrete financial management behaviors [38]. Confidence in retirement refers to beliefs and positive expectations about this stage of life [39]. Even though working population from the USA reports that, in recent years, confidence has declined notably in general terms and, specifically, also regarding retirement [40], some previous findings showed that confidence in retirement was positively related to having a specific retirement savings plan [28].

Accordingly, this work intends to analyze the relationship between confidence in retirement and FPR2, mediated by FPR1. We propose the following working hypothesis:

Hypothesis 2: Confidence in retirement will predict FPR2, and this relationship will be mediated by FPR1.

### Economic well-being

Economic well-being includes having good control of one's personal finances at the short- and mid-term, as well as dealing with unforeseen financial problems, being able to achieve financial goals, and having the economic freedom to make decisions that allow one to enjoy various aspects of life [41]. Specifically, economic well-being in retirement has been widely studied [42]. In this regard, the existence of a direct and positive relationship has been found between a family's level of income and participating in programs of financial planning [14].

Hence, the fact of having a good level of income from wages, pension funds, investments, etc. has been considered a predictor of FPR [5]. Also, having a good level of economic well-being is something that all people desire and seek, but not all succeed because economic well-being frequently depends not only on the net amount of money but also on adequate economic resource management, appropriate planning, and informed decision-making [43]. Empirical results support that households that have large debts give higher priority to paying them instead of saving for retirement, because the fact of delaying saving for retirement involves an increase in the available financial amount [44].

**Fig 1:**
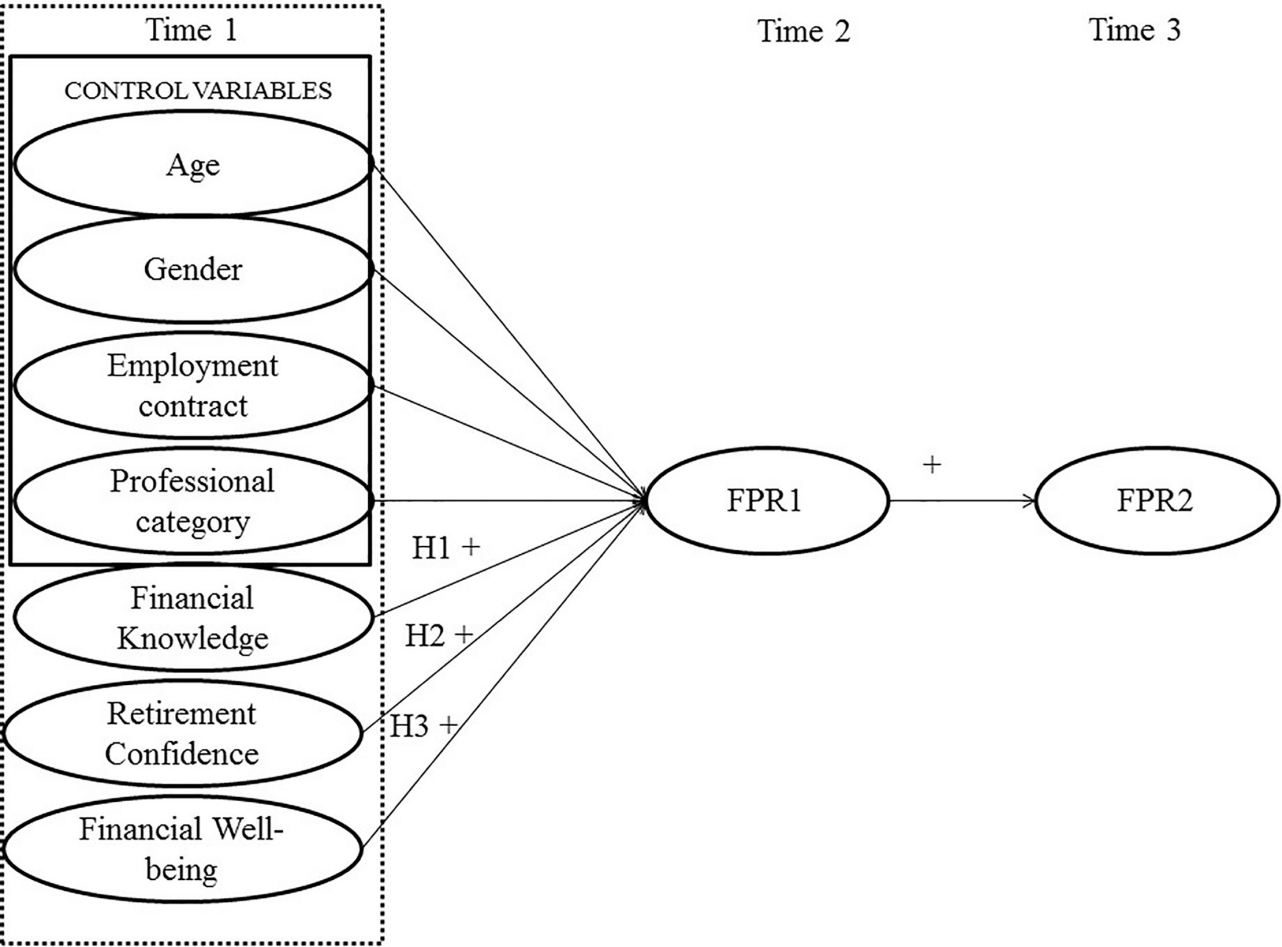
Theoretical model for the study: Antecedent variables, FPR 1 and FPR 2.

In general, it has been found that workers increase their participation in savings accounts with tax incentives depending on their age and income level [45], which supports the positive relationship between economic well-being and FPR. Lastly, this paper aims to study the relationships between economic well-being and FPR2, mediated by FPR1. Thus, we propose the following working hypothesis:

Hypothesis 3: Economic well-being will predict FPR2, and this relationship will be mediated by FPR1. (Fig 1)

## Materials and methods

### Ethical procedures

The National Distance Education University Bio-Ethical Committee approved this research under the protocol number 4/05/2016. The only inclusion criteria in the study were being older than 45 years of age and the work situation (full time or part time active workers). Potential participants were informed about the research objectives, anonymity, voluntariness, and the possibility of leaving the study at any time. They received a consent form and, if they agreed to participate, they returned it signed.

### Participants

Research with older workers are often criticized for relying on convenience samples affected by unknown selectivity. With the aim of addressing this concern, we used a national sample of SMEs to obtain generalizable results for the population of older adults. However, this involved a further restriction to reduce the sample to the limited number of workers aged 45 and over. In the present study, the sample consisted of 269 Spanish workers aged 45–62 years. The mean age of the participants was 55.1 (*SD* = 3.3). The mean number of dependents in the household was 1 (*SD* = 1.3). Mean tenure in the company was 22 years (*SD* = 10.3). Most of the participants (73%) felt that they could retire within 6 to 10 years, approximately. The other demographic features are displayed in Table 1.

**Table 1:** Demographic characteristics of participants.

Sample Characteristic	Categories	Percentage
Gender	Males	55.8
	Females	44.2
Education Level	University	23
	High School or Lower	38.7
	Missing Values	38.3
Type of Employment Contract	Full Time	86.2
	Part-Time	13.5
	Missing Values	0.3
Professional Category	Employees	49.8
	Middle Managers	36.1
	Missing Values	14.1

In order to address concerns about potential differences among those participants who left the study and the rest that continued, we compared participants at Time 1 and Time 3 regarding three demographic characteristics (age, professional category and current employment situation). Findings of the ANOVA analyses showed that there are not statistical differences [age (*F*_(642, 1)_ = .013); professional category (*F*_(642, 1)_ = .076) and current employment situation (*F*_(642, 1)_ = .02)].

### Procedure

The study design included three measures: at Time 1, we measured the variable antecedents (controls, financial literacy, confidence in retirement, and economic well-being); three months later, at Time 2, we measured FPR1; and 3 months later, at Time 3, we measured FPR2. To collect the data, the research group emailed 60 SME’s (Small and Medium Sized Enterprises) in Spain to propose a broad study on Financial Planning for retirement. This list included some firms that have collaborated in the past with the National Distance Education University by offering paid traineeships, some firms for which ex-alumni were managers or middle managers, and other firms contacted through the personal relationships of the research group’s members. Only 38 SMEs took part in the study. Related to their main economic activities, 13% of the SMEs developed them in the industry, 5% finances, 7% health services, 9% education, 18% commerce and distribution, 4% food industry, 2% energy, 22% services in general, 3% agriculture, 5% technology and communications and 12% other activities. The total population of +45 employees in these firms were contacted. At Time 1, we distributed 500 questionnaires, of which we received 375 completed (75% response rate). At Time 2, we collected 296 questionnaires (59.2% response rate), and at Time 3, we received 274 questionnaires (54.8% response rate). Of these questionnaires, only 269 were correctly completed, which are those analyzed for this work.

### Instruments

#### Demographic information and control variables

We asked the participants about their age, gender, educational level, number of dependents at home, professional category, tenure in the company, type of employment contract, and estimated retirement age.

#### Financial literacy

We used a six-item scale to measure individuals' general financial literacy [46]. Example items of this scale are “I know a lot about financial planning for retirement” or “When I need to consult about finances, I know exactly where to get the information”. All items are rated on a 5-point response format ranging from 1 (*strongly disagree*) to 5 (*strongly agree*). This measure has shown high levels of internal consistency (α = .94) [46], and the Cronbach alpha found in this study was .82.

#### Confidence in retirement

This 5-item scale measures people's confidence to achieve their goals related to retirement [31]. Participants were asked to indicate their level of confidence associated with the following statements, among which were “You adequately prepare retirement financially,” “You have enough money to live on for the rest of your life, regardless of how long you live.” The response format was a 4-point Likert-type scale ranging from 1 (*not at all confident*) to 4 (*very confident*). The value of the Cronbach’s alpha found in this study was .90.

#### Economic well-being

This brief scale consists of 4 items and is used to measure people's subjective view of their financial situation [47]. The instructions that were given to the participants were “Tell us how satisfied you feel about the following aspects”, such as, for example, “current household income” or “money available for emergencies.” The response format was a 5-point Likert-type scale, ranging from 1 (v*ery unsatisfied*) to 5 (*very satisfied*). The value of Cronbach’s alpha in this research was .85.

#### Financial planning for retirement

Following the recommendations of some authors [48], we used two different instruments to measure FPR at Time 1 and at Time 2 (FPR1 and FPR2). The goal was to avoid the bias of common variance due to the instruments and to support that both self-report measures are not simply interpretable in artifactual terms (i.e. due to the social desirability of FPR as an evidence of control and success in our society).

At Time 1, we used the *Financial Planning for Retirement Scale* [49]. Participants completed 9 items using a 7-point scale ranging from 1 (*strongly disagree*) to 7 (*strongly agree*). The instructions required participants to focus on the financial planning activities they had carried out in the past 12 months. Example items are “I have made specific expenditure plans for the future” and “I have made voluntary contributions to a savings plan for retirement.” This measure has shown high values of internal consistency in the past (α = .87; [49]), and the value of Cronbach’s alpha found in this study was .91.

At Time 2, we used a 5-item scale of about savings from the *Financial Preparedness* subscale of Noone, Stephens, and Alpass [50] which is part of *The Process of Retirement Planning Scale* (PRePS). The participants had to express their level of agreement or disagreement with statements like “When I retire, I will have enough income to ensure my standard of living” or “If I retired today, I would have enough money to cope with my retirement.” The response format is a 5-point Likert-type ranging from 1 (*strongly disagree*) to 5 (*strongly agree*). This measure has shown appropriate values of internal consistency in the past (α = .75; [50]), and the value of Cronbach’s alpha found in this study was .80.

## Results

### Descriptive analyses and correlations

Means, standard deviations, and correlations of all the variables are shown in Table 2. FPR1 obtained a mean of 2.64 (*SD* = .81), and FPR2 a mean of 3.22 (*SD* = .73).

**Table 2:** Descriptive statistics and correlation Matrix.

Variables	M	SD	1	2	3	4	5	6	7	8	9
Control Variables											
1. Age	55.1	3.30	-								
2. Gender	-	-	-.11	-							
3. Employment contract	-	-	.07	.20**	-						
4. Professional category	-	-	-.02	.16**	.19**	-					
Predictor Variables											
5. Self-Perceived Financial knowledge	2.81	.73	-.05	-.13*	.05	.26**	.73				
6. Retirement confidence	3.37	.74	-.13*	.19*	.05	.17**	.32**	.85			
7. Economic well-being	3.16	.72	-.21**	.01	.03	.15*	.31**	.59**	.82		
Mediator Variable											
8. FPR1	2.64	.81	-.10	-.01	.08	.19**	.51**	.43**	.44**	.76	
Criterion Variable											
9. FPR2	3.22	.73	.09	.01	.03	.18**	.34**	.64**	.56**	.50**	.74

Note: N = 269. Values in italics in the diagonal are the squared root of AVE of the latent variables.

Response scale Likert type with 5 points except for retirement confidence (4 points) and FPR1 (7 points).

* p < .05

** p < .01

FPR2 was significantly and positively correlated with financial literacy (*r* = .34, *p* < .01), confidence in retirement (*r* = .64, *p* < .01), economic well-being (*r* = .56, *p* < .01), and FPR1 (*r* = .50, *p* < .01). However, it is important to note that the professional category also correlated positively and significantly with FPR2 (*r* = .18, *p* < .01), whereas age and employment status correlated positively but non-significantly with FPR2. FPR1 was also significantly and positively correlated with financial literacy (*r* = .51, *p* < .01), confidence in retirement (*r* = .43, *p* < .01), economic well-being (*r* = .44, *p* < .01), and professional category (*r* = .19, *p* < .01). Age correlated negatively, albeit non-significantly, with FPR1.

### Hypothesis testing

Data analysis was performed with the *Smart PLS* program [51] which estimates standardized regression coefficients to measure relations between the latent variables using the partial least squares technique. The significance of the relations in the structural model was considered by bootstrapping 5,000 samples of 269 cases with a critical *t* -value of 1.96 for *p* < .05. To analyze the mediation hypothesis, we used the macros for SPSS INDIRECT [52] and the *Latent Variable Scores* (non-standardized scores) generated by the *Smart PLS* program when running the INDIRECT macro. First, we evaluated the measurement model and then the structural model.

#### Measurement model

We analyzed the value of the standardized factorial loadings (λ) to determine the individual reliability of the items. All the items had λ-values equal to or higher than .60 (Table 3). Also, to assess the reliability of the measurement scales, we calculated the value of Cronbach’s alpha (Table 3), and this value was in all cases higher than the recommended value of .70 [53].

**Table 3:** Reliability and convergent validity.

Latent variable		Ítem	λ	CFC	α	AVE
Self-Perceived Financial Knowledge		CF1	.76	.87	.82	.53
		CF2	.81			
		CF3	.60			
		CF4	.79			
		CF5	.79			
		CF6	.61			
Retirement Confidence		CJ1	.87	.93	.90	.72
		CJ2	.85			
		CJ3	.87			
		CJ4	.84			
		CJ5	.81			
Economic well-being		BE1	.79	.90	.85	.68
		BE2	.82			
		BE3	.84			
		BE4	.83			
FPR1		FPR2(1)	.77	.93	.91	.58
		FPR2(2)	.82			
		FPR2(3)	.83			
		FPR2(4)	.75			
		FPR2(5)	.64			
		FPR2(6)	.70			
		FPR2(7)	.78			
		FPR2(8)	.81			
		FPR2(9)	.76			
FPR2		FPR1(1)	.81	.86	.80	.55
		FPR1(2)	.60			
		FPR1(3)	.78			
		FPR1(4)	.77			
		FPR1(5)	.81			

Note: N = 269

Convergent validity was measured using the *Average Variance Extracted*, (AVE), whose value was greater than .50 for all the constructs (Table 3). Likewise, to confirm discriminant validity, the correlations between the constructs should not be higher than .80, and the value of the square root of AVE should be greater than the correlation between the constructs [54]. In this work, both conditions were met (see Table 3).

#### Structural model

**Fig 2:**
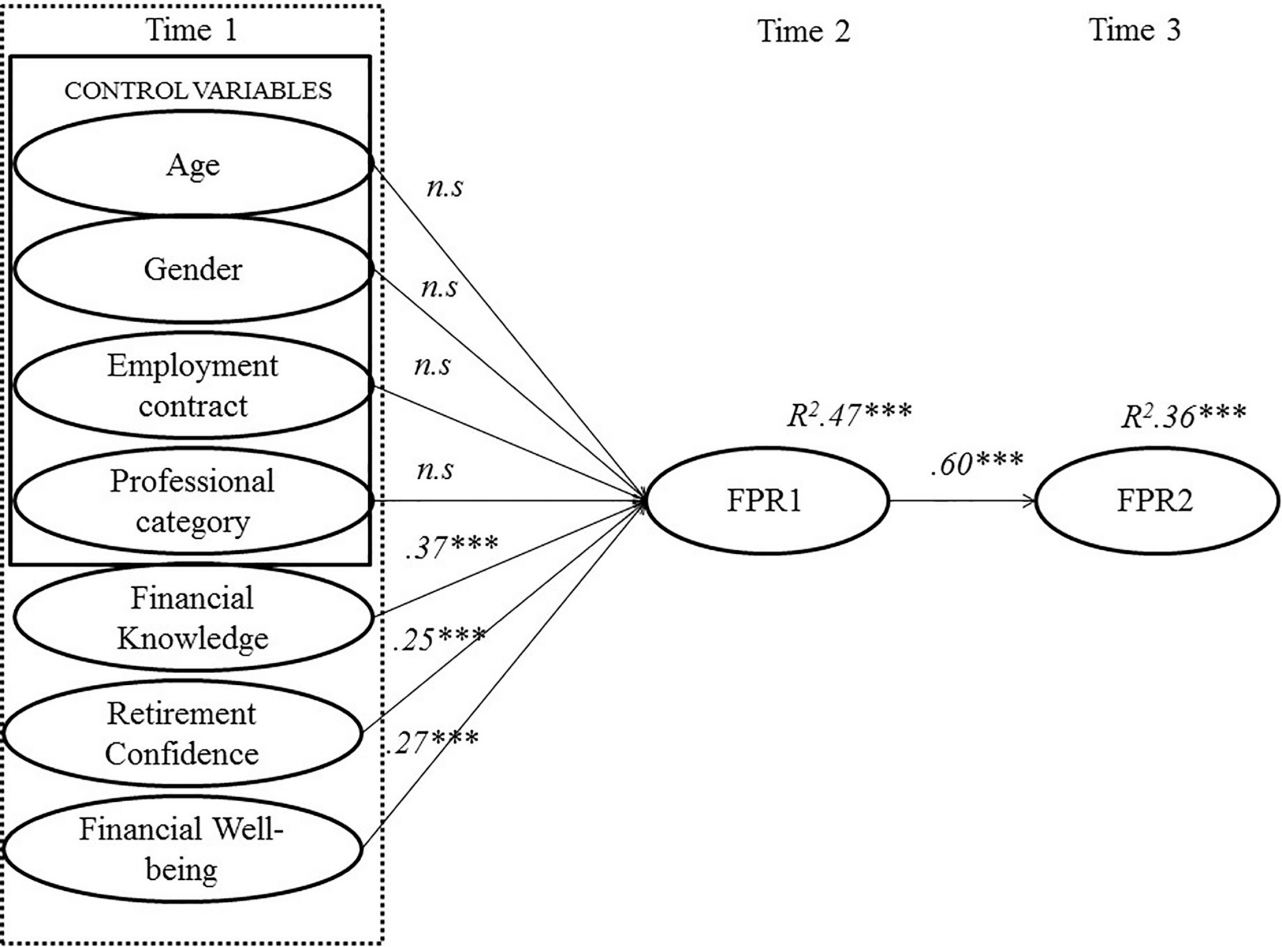
Structural model: Antecedent variables, FPR 1 and FPR 2. *Note*: *** *p* < .001. Values above the arrows are standardized regression coefficients, and values above the circles are percentages of explained variance.

The results obtained with this model show that self-perceived financial knowledge, confidence in retirement, and economic well-being had significant influence on FPR1 (Fig 2). The coefficients of the predictive variables of FPR1 were significant: financial literacy (*β* = .37, *p* < .001), confidence in retirement (*β* = . 25, *p* < .001), and economic well-being (*β* = .27, *p* < .001), accounting for 47% of the variance of FPR1 (*R*^2^ = .47). Likewise, FPR1 also had a significant influence on FPR2 (*β* = .60, *p* < .001), accounting for 36% of its variance (*R*^2^ = .36). The control variables did not have a significant influence on FPR1 (obtaining betas lower than .10): age (*β* = .01), gender (*β* = .01), professional category (*β* = .01), and employment status (*β* = .05).

To analyze the mediation hypotheses, we used the INDIRECT macro for SPSS [52]. The confidence intervals (CI) were analyzed with a confidence level of 95%, and, as they did not contain the value 0, this means that the indirect effect was significant. Thus, the results indicate that FPR1 significantly mediates between the predictors (financial literacy, confidence in retirement, and economic well-being) and FPR2, confirming working Hypotheses 1, 2, and 3 (Tables 4, 5 and 6).

**Table 4:** Direct, total and indirect effects for the mediation FPR1 among Self perceived financial knowledge and FPR2.

	B	SE	t	axb	SE	95% CI
Total Effect	.17***	.05	3.53			
Direct Effect	.08	.05	0.55			
H1: Self -perceived Financial Knowledge → FPR1→ FPR2				.09	.03	[.04, .16]

Note: N = 269; CI = Confidence interval; SE = Standard Error; Bootstrap = 5.000 samples

*** p < .001

**Table 5:** Direct, total and indirect effects for mediation FPR1 among Retirement Confidence and FPR2.

	B	SE	t	axb	SE	95% CI
Total Effect	.47***	.06	8.33			
Direct Effect	.41***	.06	7.19			
H2: Retirement Confidence → FPR1→ FPR2				.07	.02	[.03, .13]

Note: N = 269; CI = Confidence interval; SE = Standard Error; Bootstrap = 5.000 samples

*** p < .001

**Table 6:** Direct, total and indirect effects for the mediation FPR1 among economic well-being and FPR2.

	B	SE	t	axb	SE	95% CI
Total Effect	.23***	.06	3.83			
Direct Effect	.15*	.06	2.60			
H3: Financial well—being→ FPR1→ FPR2				.07	.02	[.03, .12]

Note: N = 269; CI = Confidence interval; SE = Standard Error; Bootstrap = 5.000 samples

* p < .05

*** p < .001

The results show that confidence in retirement and economic well-being have a direct effect on FPR2 (b = .41, *p* = .001 and b = .15, *p* = .01, respectively), whereas financial literacy does not show a direct effect on FPR2 (b = .08, *p* = .1226).

## Discussion

This study was aimed to analyze the relationships between the distal antecedents—financial literacy, confidence in retirement, and economic well-being—and FPR evaluated at two different time-points. First, self-perception of financial knowledge was positively related to FPR, in line with other previous works from different countries [35, 55]. We also confirmed previous results reporting that financial literacy increases the chances of participating in retirement savings plans [56]. In keeping with our results, Mullocky and Turcotte [29] revealed that people who have higher levels of financial literacy feel safer and more confident about the FRP they are carrying out.

Secondly, this study found that confidence in retirement is positively and significantly related to FPR, confirming the results of previous studies [57, 30]. Third, this study revealed that financial well-being is also positively related to FPR. Other works have pointed out that the level of income is closely related to the amount of savings for FPR [58, 59], such that individuals with a lower economic well-being save less for retirement [60], and in many cases, wages and investments are a good predictor of FPR [3]. However, Jacobs-Lawson and Hershey’s study [46] contradicts our findings, showing that people with adequate economic well-being do not plan their retirement in financial terms. Thus, despite that the causal relationships between financial resources and sufficient savings has not been firmly established, we would suggest continuing paying attention to the provision of sufficient income during retirement, specifically for some social groups that have a greater risk of poverty in advanced ages, as separated, divorced, or widowed people [61]. On another hand, the possibility of compensating the economic difficulties of many older workers is threatened by the low employment rates among these groups [62, 63, 64].

Considering the comparison among the impacts of predictors (self-perceived financial knowledge, retirement confidence and economic well-being) on FPR2, we could observe that financial knowledge has no impact, while the influence of economic well-being and specifically retirement confidence seem to be strong. First, we must recognize that our measure of financial knowledge does not include objective questions, and instead it assessed respondents’ confidence on their knowledge. So, it is likely that this measure contains a lot on “noise” which could explain the lack of significance in the direct effect. Second, the strongest impact of retirement confidence seems to agree with previous findings about that Spanish population continue to rely on public protection system as a solid support for their well-being in old age [65]. Finally, the findings about the statistically significant impact of economic well-being on FPR2 confirm previous studies even though the existing evidence on this score is controversial. Certain other studies [46] suggested that financial resources do not determine retirement saving, because people often do not save despite having sufficient financial resources to do so; while others pointed in the opposite direction [66].

Regarding the stability of FPR over time, in this work, it was found that the first step of planning, at three months, has predictive power over the second, at six months. However, it was found that FPR decreases slightly between our first and second assessments. In this regard, it is important to note the assessment of the variable by means of two different tools can introduce some source on uncontrolled variance in the results. Moreover, the potential influence of moderators in the relationship between FPR1 and FPR2, which were not explored, could also affect the findings. In this sense, although the performance of a wide range of behaviors is influenced by behavioral intention, availability of resources and opportunities—such as money, time, cooperation of other people, skills, etc.—can moderate this relationship between intention and behavior [67]. In addition, theory of planned behavior literature [68] shows that the intention to carry out a behavior, along with one’s perceptions of control over it, influences the variance of the occurrence of current behavior. In this connection, it has also been found that, when behaviors are not well learned or are carried out in unstable contexts, it is more likely for the person to have to consciously make the decision either to continue or drop these behaviors [69]. Moreover, it has been observed that the predictive power over behavior increases when this assessment is carried out in a short period of time in comparison with assessments separated by longer time intervals [70]. Therefore, in this paper, FPR1 is considered slightly greater than FPR2, that is, they present some variability over time, which can be due at least in part for the different assessment procedures.

### Limitations

The sample includes only participants from Spain and it is necessary to consider that pension systems, which maintain a close relationship with FPR, are currently in a period of constant change and vary considerably depending on the country. In addition, the sample used is small, so the described findings should be considered cautiously. An additional limitation is related to the recruitment of the sample procedure. Some authors referred this procedure as “two-stage sampling” approach [71], because we first selected a specific pool of enterprises, and then invited the actual population of workers aged 45 and over in these firms to participate. Moreover, as the majority (99.87%) of the Spanish firms are SME’s [72], we included this kind of organizations with the aim of recruiting +45 employees. Even though our data have been provided by a convenience sample, we have no reason to believe that this sample is necessarily different from the population of interest.

Our results are estimated on a sample of interest based on the total amount of +45 employees in the firms that take part in the study, instead of being a descriptive study of a representative population. Despite this fact, the variables included in our research are normally used in the literature to predict FPR and they have proven their adequacy for different cultural contexts [50, 55]. At the same time, we recognize that other potential mediators in the relationships between predictors and outcomes have been ignored, and this fact could blur our findings. Several variables come to mind, such as personality features and dispositional traits, which should be included in future studies. In this sense, patience could be a mediator variable affecting FPR1 and FPR2, such as previous studies suggested [73]. Considering that patience implies willingness to sacrifice current satisfaction for future rewards, it might result in higher preparedness for retirement. In the opposite direction, some personality traits, as need for cognitive closure can negatively affect retirement preparation as recent research showed [74]. However, another limitation to bear in mind is the data collection procedure because the use of self-reports can produce an uncontrolled source of error of the common variance.

Thus, to advance research on FPR, more objective measures should also be employed [75]. However, when performing various measures of FPR, they should be spaced in greater time intervals to determine whether this measure remains stable when working with such intervals (e.g., annual).

### Future lines of research

At the theoretical level, we underline that very diverse factors influence FPR, and the nature of relationships between psychological, financial, and social variables is tremendously complex. For this reason, is increasingly necessary to use interdisciplinary approaches to the study of retirement and its consideration from a holistic perspective [3]. However, there have been few advances in this line, and only a few specialists have progressed in this task [76].

As for a better development of the model, the influence of certain variables, such as the information received by word of mouth, the incidence of financial advisors, or the level of indebtedness of households, could be explored [44, 77]. In addition, certain psychological aspects such as the influence of the negative stereotypes associated with age may be related to attitudes toward retirement [78] and have an impact on some older workers' ability to save. In the same vein, due to the strong connection between health and wealth, financial planning for retirement should be considered in its relationship with health planning for retirement [79, 80, 81, 82]. All this should be considered, without forgetting the relevance of similar studies with larger samples and more countries that enrich and advance the study of FPR.

Finally, we could affirm that the main contribution of the present research would be related to stability of the FPR across measures, even though two different scales have been used. Despite this fact, our evidence regarding this stability is preliminary and incomplete, because the two ways of assessment of FPR were based only on self-reported information, and they slightly differ in their focus. The first measure evaluated FPR by actions taken during the previous year, while the second assessed impression about the future economic situation in retirement. As financial preparedness for retirement is a matter of growing concern, stability across different types of measures deserves further attention.

#### Implications for intervention

As a function of the proposed model, we suggest that economic, psychological, and sociological factors allow us to explain the difficulty of saving and planning for retirement, specifically for some social groups [83, 84]. Specifically, in view of the relationship between financial literacy and FPR, it seems necessary to improve the level of people's financial literacy [85]. In the same vein, financial literacy is closely related to the level of wealth, which, in turn, is strongly associated with life satisfaction [86].

### Conclusion

This study supports previous works that show that financial literacy, confidence in retirement, and economic well-being are positively related to FPR. It also supports the idea of that FPR is considerably stable over time, and that a prior measure of FPR a good predictive variable of subsequent FPR. In this way, the findings support the idea that FPR is influenced by economic, psychological, and social factors which should be considered essential to improve and develop future explanatory models of FPR.

## References

[1] Topa G, Lunceford G, Boyatzis RED. Financial planning for retirement: A psychosocial perspective. Front. Psychol. 2017; 8: 2338 10.3389/fpsyg.2017.02338 29416519 10.3389/fpsyg.2017.02338 PMC5787562

[2] Budowski M, Schief S, Sieber R. Precariousness and quality of life—a qualitative perspective on quality of life of households in precarious prosperity in Switzerland and Spain. Appl Res in Qual of Life. 2016; 11(4): 1035–1058.

[3] Rudzinska-Wojciechowska J. If you want to save, focus on the forest rather than on trees. The effects of shifts in levels of construal on saving decisions. PLoS ONE. 2017; 12(5): e0178283 10.1371/journal.pone.0178283 28552943 10.1371/journal.pone.0178283 PMC5446163

[4] Ekici T, Koydemir S. Income expectations and happiness: evidence from British panel data. Appl Res in Qual of Life. 2016;11(2): 539–552

[5] Hershey DA, Jacobs-Lawson JM, Austin JT. Effective Financial Planning for Retirement In Wang M, editor. The Oxford Handbook of Retirement. New York: Oxford University Press; 2013 pp. 402–430.

[6] Russell K, Stramoski S. Financial management practices and attitudes of dental hygienists: A descriptive study. Am Dental Hygienists Assoc. 2011; 85(4): 340–347. 22309875

[7] Helman R, Copeland C, Vanderhei J. The 2011 Retirement Confidence Survey: Confidence Drops to Record Lows, Reflecting "The New Normal" Employee Benefit Research Institute. 2011; 355: 1–39. 21542523

[8] Ward EV, Dhami MK. Editorial: The Aging Decision-Maker: Advances in Understanding the Impact of Cognitive Change on Decision-Making. Front. Psychol. 2016; 7:1622 10.3389/fpsyg.2016.01622 27818639 10.3389/fpsyg.2016.01622 PMC5073209

[9] Glamser FD, DeJong GF. The efficacy of preretirement preparations programs for industrial workers. J Gerontol. 1975; 30(5): 595–600. 10.1093/geronj/30.5.595 1181365 10.1093/geronj/30.5.595

[10] Helman R, Paladino V. Will Americans Ever Become Savers? The 14th Retirement Confidence Survey. Employee Benefit Research Institute. 2004; 268: 1–17. http://www.ebri.org/pdf/briefspdf/0404ib.pdf 15101234

[11] Grable JE, Joo S. Factors Associated with Seeking and Using Professional Retirement-Planning Help. Family and Consumer Sciences. 2001; 30(1). 37–63. 10.1177/1077727X01301002

[12] Cheung F, Yeung DY, Wu AM. Occupational future time perspective and successful aging at work. J Career Dev. 2017; 0894845317696805.

[13] Mooney A, Earl JK, Mooney CH, Bateman H. Using Balanced Time Perspective to Explain Well-Being and Planning in Retirement. Front Psychol. 2017; 8: 1781 10.3389/fpsyg.2017.01781 29081757 10.3389/fpsyg.2017.01781 PMC5646178

[14] Davies EM, Van der Heijden BI, Flynn M. Job satisfaction, retirement attitude and intended retirement age: a conditional process analysis across workers’ level of household income. Front Psychol. 2017; 8: 891 10.3389/fpsyg.2017.00891 28620329 10.3389/fpsyg.2017.00891 PMC5450519

[15] Peetz J, Simmons M, Chen Buehler R. Predictions on the go: Prevalence of spontaneous predictions for purchases. Judg Dec Making, 2016; 11(1): 48–61.

[16] Petalas DP, van Schie H, Vettehen PH. Forecasted economic change and the self-fulfilling prophecy in economic decision-making. PloS ONE. 2017; 12(3): e0174353 10.1371/journal.pone.0174353 28334031 10.1371/journal.pone.0174353 PMC5363911

[17] Kuzmanovic B, Rigoux L. Valence-Dependent Belief Updating: Computational Validation. Front. Psychol. 2017; 8:1087 10.3389/fpsyg.2017.01087 28706499 10.3389/fpsyg.2017.01087 PMC5489622

[18] Palací F, Jiménez I, Topa G. Economic cognitions among older adults: parental socialization predicts financial planning for retirement. Front Ag Neur. 2017; 9: 376. 10.3389/fnagi.2017.00376 PMC5702362 29209198

[19] Brown S, Ghosh P, Taylor K. Household Finances and Social Interaction: Bayesian Analysis of Household Panel Data. Rev Inc Wealth. 2015; 62(3): 467–488. 10.1111/roiw.12174

[20] Shoda Y, Mischel W. Personality as a stable cognitive-affective activation network: Characteristic patterns of behavior variation emerge from a stable personality structure In Read SJ, Miller LC, editors. Connectionist models of social reasoning and social behavior. Mahwah: Erlbaum; 1998; pp.175–208.

[21] Koposko JL, Hershey DA. Parental and early influences on expectations of financial planning for retirement. J Pers Fin. 2014; 13(2): 17–27.

[22] Ganzach Y, Amar M. Intelligence and the repayment of high-and low-consequences debt. Pers Indiv Diff. 2017; 110: 102–108.

[23] Gerlach P. The games economists play: Why economics students behave more selfishly than other students. PloS ONE. 2017; 12(9): e0183814 10.1371/journal.pone.0183814 28873465 10.1371/journal.pone.0183814 PMC5584942

[24] Cole S, Shastry K. Smart Money: The Effect of Education, Cognitive Ability, and Financial Literacy on Financial Market Participation. Harv Bus Sch. 2009; Working Paper 09–071.

[25] Gaurav S, Singh A. An inquiry into the financial literacy and cognitive ability of farmers: Evidence from rural India. Oxford Devel Studies. 2012; 40(3): 358–380. 10.1080/13600818.2012.703319

[26] Gariepy G, Elgar FJ, Sentenac M, Barrington-Leigh C. Early-life family income and subjective well-being in adolescents. PloS ONE. 2017; 12(7): e0179380 10.1371/journal.pone.0179380 28715418 10.1371/journal.pone.0179380 PMC5513414

[27] Hung AA, Parker AM, Yoong J. Defining and Measuring Financial Literacy. RAND Corporation—Labor and population. 2009; Working paper series WR-708. 10.2139/ssrn.1498674

[28] Lusardi A Information, expectations, and savings for retirement. In: Aaron H (editor), Behavioral Dimensions of Retirement Economics. Washington, DC: Brookings Institution Press and Russell Sage Foundation 1999; pp. 81–115.

[29] Lusardi A, Mitchell OS. The economic importance of financial literacy: Theory and evidence. J Econ lit. 2014; 52(1), 5–44 10.1257/jel.52.1.5 28579637 10.1257/jel.52.1.5 PMC5450829

[30] Mullock K, Turcotte J. Financial literacy and retirement saving. Work paper 2012–01. Department of Finance Canada. 2012; http://www.fin.gc.ca/pub/pdfs/wp2012-01e.pdf

[31] Joo S, Grable JE. Employee education and the likelihood of having a retirement savings program. Fin Couns Plan. 2005; 16(1): 37–50.

[32] Ekerdt DJ, Hackney JK. Workers' ignorance of retirement benefits. Gerontol. 2002; 42(4): 543–551. 10.1093/geront/42.4.543 10.1093/geront/42.4.543 12145382

[33] Grable JE, Lytton RH. Determinants of retirement savings plan participation: A discriminant analysis. Pers Finan Worker Prod. 1997; 1(1): 184–189.

[34] Hastings JS, Madrian BC, Skimmyhorn WL. Financial literacy, financial education and economic outcomes. An Rev Econ. 2013; 5(1): 347–373. 10.3386/w18412 10.1146/annurev-economics-082312-125807 PMC3753821 23991248

[35] Kalmi P, Ruuskanen O. Financial Literacy and Retirement Planning in Finland. 2016. http://www.taloustieteellinenyhdistys.fi/wp-content/uploads/2016/03/kalmi_ruuskanen.pdf

[36] Kimball MS, Shumway T. Investor sophistication and home bias, diversification and employer stock puzzles. Soc Sc Res Net. 2010 http://www-personal.umich.edu/~mkimball/keio/z-Osaka-2007-after/sophist1.pdf

[37] Zebrowitz LA, Boshyan J, Ward N, Gutchess A, Hadjikhani N. The older adult positivity effect in evaluations of trustworthiness: emotion regulation or cognitive capacity? PloS ONE. 2017; 12(1): e0169823 10.1371/journal.pone.0169823 28060919 10.1371/journal.pone.0169823 PMC5218557

[38] Guo L, Stone D, Bryant S, Wier B, Nikitkov A, Ren C, Riccio EL, Shen M, Trabelsi S, Zhang L. Are consumers' financial needs and values common across cultures? Evidence from six countries. Int J Cons St. 2013; 37(6): 675–688. 10.1111/ijcs.12047

[39] Kim J, Kwon J, Anderson EA. Factors related to retirement confidence: Retirement preparation and workplace financial education. Fin Couns Plan. 2005; 12(2): 77–89.

[40] Helman R, Greenwald M, Adams N, Copeland C, Van Derhei J. The 2013 retirement confidence survey: Perceived savings needs outpace reality for many. 2013 http://www.ebri. org/files/Final-FS.RCS-13.FS_3.Saving.FINAL.pdf 23678670

[41] Consumer Financial Protection Bureau. Financial Well-Being: The Goal of Financial Education. Report, Iowa City, IA: 2015.

[42] Plouffe RA, Tremblay PF. The relationship between income and life satisfaction: Does religiosity play a role? Pers Ind Diff. 2017; 109: 67–71.

[43] Drever AI, Odders-White E, Kalish CW, Else-Quest NM, Hoagland EM, Nelms EN. Foundations of Financial Well-Being: Insights into the Role of Executive Function, Financial Socialization, and Experience-Based Learning in Childhood and Youth. J Cons Aff. 2015; 49(1): 13–38. 10.1111/joca.12068

[44] Bernstein D. Household debt and IRAs: evidence from the Survey of Consumer Finances. Fin Couns Plan. 2004; 15: 63–72.

[45] Springstead GR, Wilson TM. Participation in voluntary individual savings accounts: An analysis of IRAs, 401(k)s, and the TPS. Soc Sec Bull. 2000; 63: 34–39. 10951688

[46] Jacobs-Lawson JM, Hershey DA. Influence of future time perspective, financial knowledge, and financial risk tolerance on retirement saving behaviors. Fin Serv Rev. 2005; 14(1): 331–344.

[47] Hayhoe CR, Wilhelm MS. Discriminating between primary family financial managers and other adults in the family. Fin Couns Plan. 1995; 6(1): 75–82.

[48] Ellis MV. Repeated measures designs. The Counsel Psychol. 1999, 27, 4, p. 552–578.

[49] Stawski RS, Hershey DA, Jacobs-Lawson JM. Goal clarity and financial planning activities as determinants of retirement savings contributions. Int J Ag Human Devel. 2007; 64(1): 13–32. 10.2190/13GK-5H72-H324-16P2 17390963

[50] Noone JH, Stephens C, Alpass F. The Process of Retirement Planning Scale (PRePS): Development and Validation. Psychol Assess. 2010; 22(3): 520–531. 10.1037/a0019512 20822264 10.1037/a0019512

[51] Ringle CM, Wende S, Will S. SmartPLS (Software). Germany. 2005. http://www.smartpls.de

[52] Preacher KJ, Hayes AF. SPSS macro for multiple mediation (INDIRECT) (Software). Ohio State University 2008

[53] Nunnally JC. Psychometric theory. New York, NY: McGraw-Hill 1978.

[54] Chin WW. The partial least squares approach to structural equation modeling In Marcoulides GA, editor, Modern methods for business research. Mahwah: Erlbaum; 1998 pp. 295–336.

[55] Mansor MF, Hong CC, Abu NH, Shaari MS. Demographic factors associated with retirement planning: A study of employees in Malaysian Health Sectors. As Soc Sc. 2015; 11(13): 108–116. 10.5539/ass.v11n13p108

[56] Topa G, Segura A, Pérez S. Gender differences in retirement planning: a longitudinal study among Spanish Registered Nurses. J Nurs Manag. 2018 10.3389/fpsyg.2018.00410 10.1111/jonm.12586 29464800

[57] Moorthy MK, Chelliah TD, Chiau SS, Lai CL, Ng ZK, Wong CR, Wong YT. A study on the retirement planning behavior of working individuals in Malaysia. Int J Acad Res Econ Manag Sc. 2012; 1(2): 54–72.

[58] Fisher PJ, Anong ST. Relationship of saving motives to saving habits. J Fin Couns Plan. 2012; 23(1): 63–79.

[59] Hira TK, Rock WL, Loibl C. Determinants of retirement planning behavior and differences by age. Int J Cons Studies. 2009; 33(3): 293–301. 10.1111/j.1470-6431.2009.00742.x

[60] DeVaney SA, Zhang TC. A cohort analysis of the amount in defined contribution and individual retirement savings. Fin Couns Plan. 2001; 12(1): 89–104.

[61] Bütler M, Huguenin O, Teppa F. What Triggers Early Retirement? Results from Swiss Pension Funds., Centre for Economic Policy Research. 2004; Working Paper 4394.

[62] Derous E, Decoster J. Implicit age cues in resumes: subtle effects on hiring discrimination. Front Psychol. 2017; 8: 1321 10.3389/fpsyg.2017.01321 28848463 10.3389/fpsyg.2017.01321 PMC5554369

[63] Dordoni P, Van der Heijden B, Peters P, Kraus-Hoogeveen S, Argentero P. Keep Up the Good Work! Age-Moderated Mediation Model on Intention to Retire. Front Psychol. 2017; 8: 1717 10.3389/fpsyg.2017.01717 29089905 10.3389/fpsyg.2017.01717 PMC5651082

[64] Sousa-Ribeiro M, Sverke M, Coimbra JL, De Witte H. Intentions to Participate in Training Among Older Unemployed People: A Serial Mediator Model. J Car Devel. 2017; 0894845316687669.

[65] Topa G, Segura A, Pérez S, Gender differences in retirement planning: a longitudinal study among Spanish Registered Nurses. J Nurs Manag. 2018; 26(5), 587–596. 10.1111/jonm.12586 29464800

[66] Jiménez I, Chiesa R, Topa G, Financial Planning for retirement: Age-related Differences among Spanish workers. J Career Dev. 2018; On line first.

[67] Ajzen I. The Theory of Planned Behavior. Org Beh Hum DecProc. 1991; 50(2): 179–211. 10.1016/0749-5978(91)90020-T

[68] Vo PT, Bogg T Testing Theory of Planned Behavior and Neo-Socioanalytic Theory models of trait activity, industriousness, exercise social cognitions, exercise intentions, and physical activity in a representative U.S. sample. Front. Psychol. 2015); 6:1114 10.3389/fpsyg.2015.01114 26300811 10.3389/fpsyg.2015.01114 PMC4526790

[69] Ouellete JA, Wood W. Habit and intention in everyday life: The multiple processes by which past behavior predicts future behavior. Psychol Bull. 1998; 124(1): 54–74. 10.1037/0033-2909.124.1.54

[70] McEachan RRC, Conner M, Taylor NJ, Lawton R.J. Prospective prediction of health-related behaviours with the Theory of Planned Behaviour: a meta-analysis. Health Psychol Rev. 2011; 5(2): 97–144 10.1080/17437199.2010.521684

[71] Van Solinge H. Adjustment to retirement In Wang M. (Ed.), The Oxford Handbook of Retirement, 2013 (pp. 311–324). New York: Oxford University Press.

[72] Spanish Ministry of Economy, Industry and Competitiveness. SMEs Statistics, March 2018, 16. Available at: http://www.ipyme.org/publicaciones/estadisticas-pyme-2017.pdf

[73] Arfer KB, Luhmann CC Time-Preference Tests Fail to Predict Behavior Related to Self-control. Front. Psychol. 2017, 8:150 10.3389/fpsyg.2017.00150 28232810 10.3389/fpsyg.2017.00150 PMC5298954

[74] Topa G., Hernández M. Zappalà S. Financial Management behavior among young adults: The role of Need for Cognitive Closure in a three-wave moderated mediation model. 2018 Front. Psychol. 10.3389/fpsyg.2018.02419 PMC6283974 30555400

[75] Bönte W, Lombardo S, Urbig D. Economics meets psychology: experimental and self-reported measures of individual competitiveness. Pers Indiv Diff. 2017; 116: 179–185.

[76] Dulebohn JH. An investigation of the determinants of investment risk behavior in employer-sponsored retirements plans. J Manag. 2002; 28(1): 3–26.

[77] Tauni MZ, Fang HX, Iqbal A. The role of financial advice and word-of-mouth communication on the association between investor personality and stock trading behavior: Evidence from Chinese stock market. Pers Indiv Diff. 2017; 108: 55–65.

[78] Armenta BM, Stroebe K, Scheibe S, Postmes T, Van Yperen N. W. Feeling younger and identifying with older adults: Testing two routes to maintaining well-being in the face of age discrimination. PloS ONE. 2017; 12(11): e0187805 10.1371/journal.pone.0187805 29117257 10.1371/journal.pone.0187805 PMC5678732

[79] Erreygers G.; Kessels R. Socioeconomic Status and Health: A New Approach to the Measurement of Bivariate Inequality. Int. J. Environ. Res. Public Health 2017, 14, 673. 10.3390/ijerph14070673 PMC5551111 28644405

[80] Chan B.C.L.; Luciano M.; Lee B. Interaction of Physical Activity and Personality in the Subjective Wellbeing of Older Adults in Hong Kong and the United Kingdom. Behav. Sci. 2018, 8, 71. 10.3390/bs8080071 PMC6116082 30082661

[81] Zhang W.; Meng H.; Yang S.; Luo H.; Liu D. Changes in Hypertension-Related Knowledge and Behavior and Their Associations with Socioeconomic Status among Recently Urbanized Residents in China: 2013–2016. Int. J. Environ. Res. Public Health 2018, 15, 1701. 10.3390/ijerph15081701 PMC6121690 30096907

[82] Dovie D.A. Leveraging Healthcare Opportunities for Improved Access among Ghanaian Retirees: The Case of Active Aging. Soc. Sci. 2018, 7, 92.

[83] Palmer E.K. Structural Disadvantage: Evidence of Gender Disparities in the Norwegian Pension System. Soc. Sci. 2017, 6, 22.

[84] Hoebel J.; Rommel A.; Schröder S.L.; Fuchs J.; Nowossadeck E.; Lampert T. Socioeconomic Inequalities in Health and Perceived Unmet Needs for Healthcare among the Elderly in Germany. Int. J. Environ. Res. Public Health 2017, 14, 1127. 10.3390/ijerph14101127 PMC5664628 28954436

[85] Hu N. The Misunderstanding of Social Insurance: The Inadequacy of the Basic Pension Insurance for Urban Employees (BPIUE) for the Aging Population of China. Soc. Sci. 2018, 7, 79.

[86] Gere J, Schimmack U. Benefits of income: Associations with life satisfaction among earners and homemakers. Pers Ind Diff. 2017; 119: 92–95.

